# The impact of myocardial fibrosis biomarkers in a heart failure population with atrial fibrillation—The HARVEST-Malmö study

**DOI:** 10.3389/fcvm.2022.982871

**Published:** 2022-10-19

**Authors:** Zainu Nezami, Hannes Holm, Marcus Ohlsson, John Molvin, Johan Korduner, Erasmus Bachus, Amir Zaghi, Anna Dieden, Pyotr G. Platonov, Amra Jujic, Martin Magnusson

**Affiliations:** ^1^Department of Internal Medicine, Sweden Lund University, Skane University Hospital, Lund, Sweden; ^2^Department of Clinical Sciences, Lund University, Malmö, Sweden; ^3^Department of Cardiology, Lund University, Skane University Hospital, Malmö, Sweden; ^4^Department of Clinical Sciences, Lund University, Lund, Sweden; ^5^Wallenberg Center for Molecular Medicine, Lund University, Lund, Sweden; ^6^Hypertension in Africa Research Team (HART), North West University Potchefstroom, Potchefstroom, South Africa

**Keywords:** heart failure, biomarkers, fibrosis, echocardiography, atrial fibrillation

## Abstract

**Background:**

Several studies suggest that circulating biomarkers of myocardial fibrosis are associated with worse prognosis in subjects with atrial fibrillation (AF). Here, we aimed to explore associations between fibrosis biomarkers, prevalent AF, and left atrial volume (LAV) enlargement in subjects with heart failure (HF). Additionally, we evaluated the prognostic impact of fibrotic biomarkers in HF with co-existing AF.

**Materials and methods:**

Patients hospitalized for HF (*n* = 316, mean age 75 years; 30% women) were screened for AF. Seven proteins previously associated with myocardial fibrosis [metalloproteinase inhibitor 4 (TIMP-4), suppression of tumorigenicity 2 (ST-2), galectin-3 (GAL-3), growth/differentiation factor-15 (GDF-15), and matrix metalloproteinase 2, 3, and 9 (MMP-3, MMP-3, and MMP-9, respectively)] were analyzed using a proximity extension assay. Proteins with significant Bonferroni-corrected associations with mortality and re-hospitalization risk were taken forward to multivariable Cox regression analyses. Further, Bonferroni-corrected multivariable logistic regression models were used to study associations between protein plasma levels, prevalent AF, and severely enlarged left atrial volume index (LAVI ≥ 48 ml/m^2^).

**Results:**

Prevalent AF was observed in 194 patients at the hospitalization of whom 178 (92%) were re-hospitalized and 111 (57%) died during the follow-up period. In multivariable logistic regression models, increased plasma levels of TIMP-4, GDF-15, and ST-2 were associated with the prevalence of AF, whereas none of the seven proteins showed any significant association with severely enlarged LAVI. Increased plasma levels of five proteins yielded significant associations with all-cause mortality in patients with co-existing AF; TIMP-4 (HR 1.33; CI95% 1.07–1.66; *p* = 0.010), GDF-15 (HR 1.30; CI95% 1.05–1.62; *p* = 0.017), GAL-3 (HR 1.29; CI95% 1.03–1.61; *p* = 0.029), ST-2 (HR 1.48; CI95% 1.18–1.85; *p* < 0.001), and MMP-3 (HR 1.33; CI95% 1.09–1.63; *p* = 0.006). None of the proteins showed any significant association with re-hospitalization risk.

**Conclusion:**

In this study, we were able to demonstrate that elevated levels of three plasma proteins previously linked to myocardial fibrosis are associated with prevalent AF in a HF population. Additionally, higher levels of five plasma proteins yielded an increased risk of mortality in the HF population with or without co-existing AF.

## Introduction

Atrial fibrillation (AF) is the most common cardiac arrhythmia worldwide and a leading risk factor for morbidity and mortality, thus representing a high burden to affected patients and the healthcare system (1). AF often co-exists with heart failure which constitutes a challenging dilemma for clinicians since the occurrence of both conditions aggravates each other and is associated with worse prognosis (2). In this aspect, the interest in finding new pathophysiological links between AF and HF has emerged, where growing evidence suggests that myocardial fibrosis is a contributing factor for both AF and HF development (3, 4). By its contribution to cardiac remodeling, myocardial fibrosis subsequently leads to declined cardiac relaxation and contractility (5) and constitutes an atrial substrate associated with increased left atrial volume (LAV) and development of AF (6). Thus, it is well-known that each of these conditions can be either the cause or consequence of the other (7). In recent studies, several blood-based biomarkers have been suggested as markers of the fibrotic cardiac process seen in patients with AF (8). Furthermore, many of these biomarkers including transforming growth factor β 1 (TGF-β1) have also been implicated in the pathology and prognosis of HF (9). However, there is a lack of knowledge about whether biomarkers that reflect the fibrotic process within the heart are associated with the prognosis of individuals with HF and co-existing AF. The use of myocardial fibrosis biomarkers in day-to-day clinical practice can potentially bring a better understanding of the mutual pathophysiology between AF and HF. Thus, here we aim to explore if plasma levels of myocardial fibrotic proteins are associated with the prevalence of AF and enlarged LAV in a HF population as well as to explore the prognostic impact of each protein in regard to incident mortality and re-hospitalization risk in HF patients with and without AF.

## Materials and methods

### Study population

**FIGURE 1:**
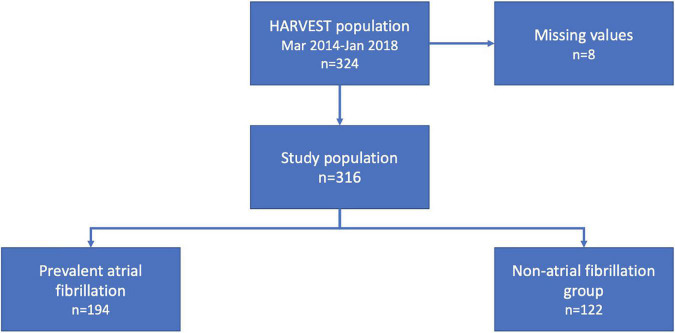
Flowchart of the study population.

The HeARt and Brain Failure inVESTigation study (HARVEST) is a prospective study undertaken in patients hospitalized for the diagnosis of HF (ICD-10: I50-) at Skane University Hospital, Sweden (10). Admission to the department of internal medicine or cardiology for the treatment of newly diagnosed or exacerbated chronic HF is the inclusion criteria for the HARVEST study. Participants who are unable to deliver informed consent are excluded. In cases of severe cognitive impairment, the relatives are informed and asked for permission on the patient’s behalf. Between 20 March 2014 and 22 January 2018, a total of 324 consecutive patients were included and underwent a clinical examination. These participants had consecutively from the study start until January 2018, been analyzed with a proximity extension assay consisting of 92 proteins. Eight patients had missing values on relevant co-variates, rendering a study population of 316 eligible participants with the complete dataset. Within the study population, 194 patients were diagnosed with co-existing AF (Figure 1). The Ethical Review Board at Lund University, Sweden has approved the study and it fulfills the Declaration of Helsinki. Written informed consent was obtained from all participants or relatives as described above.

### Co-variates

Anthropometric measurements and blood samples were obtained after an overnight fast.

Body mass index (BMI) was calculated as kilograms per square meter, and data regarding the study participants’ medication were collected. Prevalent diabetes was defined as a self-reported diagnosis of type 2 diabetes, the use of antidiabetic medication, or fasting plasma glucose >7 mmol/L. Prevalent smoking status was self-reported as yes or no, where never and previous smokers were regarded as non-smokers, and present-day smokers were defined as smokers. Systolic and diastolic blood pressures (BPs) were measured by trained nurses using a validated automated BP monitor Boso Medicus (Bosch + Sohn GmbH u. Co. KG, Jungingen, Germany). The upper arm cuff of appropriate size was placed on the right side, and the arm was supported at the heart level. Hypertension was defined as either systolic blood pressure (SBP) ≥140 mmHg and/or diastolic blood pressure (DBP) ≥90 mmHg. Atrial fibrillation (AF) was defined as the presence of AF on an electrocardiogram at the time of hospitalization or history of AF according to the patient’s medical documentation. The use of anticoagulation medication was defined as receiving treatment with either Warfarin or novel oral anticoagulants (NOACs).

### Proteomic profiling

A proximity extension assay (PEA) technique using the Proseek Multiplex CVD III 96 × 96 reagents kit (Olink Bioscience, Uppsala, Sweden) was used to measure plasma levels of 92 CVD proteins (11). The PEA technique uses two oligonucleotide-labeled specific antibodies to bind to each target protein, which allows the formation of a polymerase chain reaction sequence that can then be detected and quantified. All data are presented as arbitrary units. The CVD III panel includes 92 proteins, with established or proposed associations with metabolism, inflammation, and CVD. Across all assays, the mean intra-assay and inter-assay variations were observed to be 8.1 and 11.4%, respectively. Additional information regarding the assays is available on the Olink homepage.^1^ Seven proteins included in the CVD III panel were selected to be part of further analyses aiming to explore the cross-sectional relationship between prevalent AF and severely enlarged LAV as well as the prognostic impact of each protein for participants with and without AF. The selected proteins have been previously associated with myocardial fibrosis, metalloproteinase inhibitor 4 (TIMP-4) (12), suppression of tumorigenicity 2 (ST-2) (13), galectin-3 (GAL-3) (14), growth/differentiation factor-15 (GDF-15) (15), and matrix metalloproteinase 2, 3, and 9 (MMP-2, MMP-3, and MMP-9, respectively) (16, 17).

### Echocardiography

Transthoracic echocardiograms were available in 237 (75%) study participants and obtained using a Philips IE33 (Philips, Andover, MA, USA) with a 1–5 MHz transducer (S5-1), or with a GE Vingmed Vivid 7 Ultrasound (GE, Vingmed Ultrasound, Horten, Norway) with a 1–4 MHz transducer (M3S). All studies were performed by experienced sonographers as a part of the clinical routine at a central echocardiographic laboratory. Measurements from the parasternal long axis, apical four- and two-chamber views were done offline using Xcelera 4.1.1 (Philips Medical Systems, Netherlands) according to the recommendations of the American Society of Echocardiography. The parasternal long-axis view was used to measure internal left and right ventricular dimensions at end-diastole. Measurements of wall thickness were obtained in a two-dimensional end-diastolic parasternal long-axis view. The Simpson method was used to calculate left ventricular volumes by manual tracing (papillary muscles included in the cavity) in two-dimensional end-diastolic and end-systolic frames defined as the largest and smallest left ventricular cavities, respectively, in apical four- and two-chamber projections. The ejection fraction (EF) was calculated automatically from end-diastolic volumes (EDV) and end-systolic volume (ESV) using the following formula: EF = (EDV-ESV)/EDV. Tricuspid annular plane systolic excursion (TAPSE) and pulsed tissue Doppler (DTI)-derived tricuspid annular systolic velocity (S’) were used to measure right ventricular systolic function. M-mode images in apical four-chamber view, with the cursor optimally aligned along the direction of the tricuspid annulus, were used to obtain TAPSE. To assess S’-wave velocity, pulsed DTI images were obtained in an apical four-chamber view on the free-wall side of the right ventricle, with the basal segment and the annulus aligned with the Doppler cursor. The LA endocardial borders were manually traced in both apical four-chamber and two-chamber views. For the assessment of left atrium (LA) volumes, the biplane area-length method was used: LA volume = (0.85 × area apical four-chamber × area apical two-chamber)/(shortest atrial length). The values were indexed to BSA and defined as left atrial volume index (LAVI). Severely enlarged atrial volume was defined as LAVI ≥ 48 ml/m^2^ (18).

### Primary endpoints

The primary endpoints were all-cause mortality and the first post-discharge hospitalization. All-cause mortality was defined as death by any cause and was retrieved from the National Board of Health and Welfare’s Cause of Death Register. Re-hospitalizations defined as the first of any re-admission to the hospital were retrieved from electronic medical charts (Melior, Siemens Health Services, Solna, Sweden). All subjects were followed from study inclusion until 31 December 2020.

### Statistics

Group differences in continuous variables between study participants with or without prevalent AF were compared using a one-way ANOVA test, whereas categorical variables were compared using Pearson’s chi-square test. The variables are presented as means (standard deviation (SD)) and medians (25–75 interquartile range). All analyses were performed using SPSS Windows version 25.0 and R version 4.0.4, and a *p*-value of < 0.05 was considered statistically significant.

### Analyses of proteins associations with prevalent atrial fibrillation and left atrial volume index

In 316 subjects with a complete dataset on all co-variates, unadjusted logistic regressions were carried out exploring associations between the seven proteins and prevalence of AF. Proteins that presented with significant Bonferroni-corrected associations with prevalent AF were further adjusted for age and sex (Model 1). Proteins with significant associations with prevalent AF in Model 1 were further adjusted according to Model 2 [prevalent diabetes, current smoking, BMI, systolic blood pressure (SBP), New York heart association classification (NYHA class), anticoagulation treatment, and prior HF]. To analyze the associations of the seven myocardial fibrosis proteins with the severely enlarged atrial volume defined as LAVI ≥48 ml/m^2^, the same statistical procedure as described above was performed. Pearson’s correlation analysis was performed to explore correlations between the levels of proteins in the whole population.

### Survival analyses

In the next set of analyses, associations between the seven proteins and (1) mortality and (2) re-hospitalization risk were explored using unadjusted Cox regression analyses in three groups; all patients, subjects with and without prevalent AF. Associations that were Bonferroni-corrected significantly associated with (1) mortality and/or (2) re-hospitalization risk were further adjusted according to Model 1 (age and sex). Associations that were significant in Model 1 were further adjusted according to Model 2 (prevalent diabetes, current smoking, BMI, SBP, NYHA class, prior HF, and anticoagulation treatment). In Cox regression models including all patients, prevalent AF was added as a confounder in Model 2.

## Results

### Patient characteristics

The study population had a mean age of 75 (±12) years and was predominantly male participants (70%; *n* = 220) (Table 1). More than half of the study population had known AF diagnosed by the time of index admission, i.e., prevalent AF (61%; *n* = 194). Of these, 83% (*n* = 161) had at baseline ongoing treatment with an anticoagulant agent. Patients with co-existing AF were more likely to be older, and have lower BMI and lower SBP compared to individuals without AF. The prevalence of diabetes, current smoking, or level of NYHA class at admission did not differ between the groups (Table 1). Except for GAL-3 and MMP-9, patients with AF had higher levels of proteins associated with myocardial fibrosis as compared to those without co-existing AF. Correlations between the proteins are shown in Supplementary Table 1.

**TABLE 1:** Characteristics of study participants (*n* = 316) at baseline stratified according to the prevalence of atrial fibrillation.

Baseline characteristic	Whole population	Prevalent atrial fibrillation	Non-Atrial fibrillation group	*p*-value
	*n* = 316	*n* = 194	*n* = 122	
Age [years (SD)]	75 (12)	77 (10)	71 (13)	<0.001
Sex [female *n* (%)]	96 (30)	57 (29)	39 (32)	0.706
NYHA-class III–IV [*n* (%)]	270 (85)	170 (88)	100 (82)	0.185
Current smoking [*n* (%)]	39 (12)	20 (10)	19 (16)	0.218
BMI [kg/m^2^ (SD)]	28 (6)	27.3 (5.4)	28.6 (6.5)	0.046
SBP [mmHg (SD)]	136 (27)	134 (25)	140 (30)	0.037
DBP [mmHg (SD)]	79 (16)	79 (15)	80 (17)	0.351
Prevalent diabetes [*n* (%)]	117 (37)	69 (36)	48 (39)	0.550
Anticoagulation treatment [*n* (%)]	197 (62)	161 (83)	36 (29)	<0.001
**Antihypertensive treatment**				
ACEi [*n* (%)]	170 (54)	102 (53)	68 (56)	0.643
ARBs [*n* (%)]	81 (26)	49 (25)	32 (26)	0.895
Beta blocker [*n* (%)]	279 (88)	172 (89)	107 (88)	0.858
Diuretics [*n* (%)]	304 (96)	189 (97)	115 (94)	0.225
Aldosteron receptor antagonists [*n* (%)]	93 (29)	56 (29)	37 (30)	0.439
Prior heart failure [*n* (%)]	203 (64)	136 (70)	67 (55)	0.023
New onset of myocardial infarction [*n* (%)]	21 (7)	14 (7)	7 (6)	0.651
Prior myocardial infarction [*n* (%)]	123 (39)	73 (38)	50 (41)	0.635
Ejection fraction [% (SD)], (*n* = 237)	39 (16)	40 (15)	37 (17)	0.105
HFrEF [*n*; (%)]	123 (39)	61 (45)	62 (51)	0.017
HFmrEF [*n*; (%)]	43 (14)	29 (22)	14 (12)	0.125
HFpEF [*n*; (%)]	71 (23)	45 (33)	26 (21)	0.192
LAVI [ml/m^2^ (SD)], (*n* = 234)	54.9 (20)	59.8 (22)	48.2 (16)	<0.001
LAVI ≥ 48 ml/m^2^ [*n* (%)]	134 (42)	89 (66)	45 (45)	0.001
ST-2 (SD)	5.59 (0.93)	5.71 (0.86)	5.41 (0.99)	0.005
MMP-2 (SD)	4.43 (0.58)	4.52 (0.58)	4.28 (0.59)	<0.001
MMP-9 (SD)	4.82 (1.01)	4.70 (1.0)	4.99 (1.0)	0.016
MMP-3 (SD)	7.11 (1.01)	7.21 (0.95)	6.95 (1.08)	0.025
GAL-3 (SD)	5.99 (0.52)	5.99 (0.50)	5.98 (0.55)	0.907

NYHA class, New York Heart Association, BMI, body mass index; SBP, systolic blood pressure; DBP, diastolic blood pressure; HFrEF, heart failure with reduced ejection fraction; HFmrEF, heart failure with mildly reduced ejection fraction; HFpEF, heart failure with preserved ejection fraction; LAVI, left atrial volume index; ACEi, Angiotensin-converting enzyme inhibitors; ARBs, angiotensin receptor blockers; TIMP-4, metalloproteinase inhibitor 4; ST-2, suppression of tumorigenicity 2; GAL-3, galectin-3; GDF-15, growth/differentiation factor-15; MMP-2, 3, and 9, matrix metalloproteinase 2, 3, and 9.

## Association between atrial fibrillation, atrial size, and fibrosis biomarkers

In multivariable logistic regression models (*Model 2*), increased plasma levels of TIMP-4 (OR 1.58; CI95% 1.15–2.18; *p* = 0.005), ST-2 (OR 1.42; CI95% 1.06–1.91; *p* = 0.020), and GDF-15 (OR 1.40; CI95% 1.01–1.94; *p* = 0.046) were associated with prevalent AF (Table 2). In a sub-analysis stratified according to gender, increased plasma levels of TIMP-4 and ST-2 were associated with prevalent AF in male participants (Supplementary Table 2). In female participants, none of the proteins showed any significant association with prevalent AF. In multivariable logistic regression models (*Model 2*), none of the proteins were associated with severely enlarged LAVI (≥48 ml/m^2^) (Supplementary Table 3).

**TABLE 2:** Logistic regression analysis examining proteins association with prevalent atrial fibrillation.

	Unadjusted			Model 1			Model 2		
Proteins	OR	95%CI	*p*-value	OR	95%CI	*p*-value	OR	95%CI	*p*-value
TIMP-4	1.70	1.33–2.18	3.1 × 10^–5^	1.56	1.19–2.03	0.001	1.58	1.15–2.18	0.005
ST-2	1.40	1.10–1.78	0.006	1.36	1.07–1.74	0.013	1.42	1.06–1.91	0.020
MMP-2	1.54	1.21–1.95	4.9 × 10^–4^	1.53	1.19–1.97	0.001	1.32	0.97–1.78	0.075
GDF-15	1.59	1.24–1.04	2.9 × 10^–4^	1.42	1.10–1.83	0.007	1.40	1.01–1.94	0.046
GAL-3	1.01	0.81–1.27	0.906	–	–	–	–	–	–
MMP-9	0.75	0.60–0.95	0.017	–	–	–	–	–	–
MMP-3	1.31	1.03–1.65	0.026	–	–	–	–	–	–

TIMP-4, Metalloproteinase inhibitor 4; ST-2, suppression of tumorigenicity 2; GAL-3, galectin-3. Matrix metalloproteinase 2, 3, and 9 (MMP-2, MMP-3, and MMP-9, respectively). Model 1: age and sex. Model 2: age, sex, body mass index, systolic blood pressure at admission, prevalence of diabetes, prior heart failure, current smoking, anticoagulation treatment, and New York heart association class (NYHA class) as independent variables.

### Association between fibrosis biomarkers, mortality, and re-hospitalization risk

During the follow-up period (March 2014 to January 2018), a total of 277 (88%) were re-hospitalized and 177 (56%) patients died. The median follow-up time to hospital admission and death was 136 [interquartile range (IQR) 533] and 1,180 (IQR 1178) days, respectively. Of these cases, 178 (64%) and 111 (63%) had AF, respectively. In the whole population, increased levels of six proteins were associated with mortality; TIMP-4, GDF-15, GAL-3, ST-2, MMP-2, and MMP-3. For re-hospitalization risk within the whole population, increased levels of TIMP-4 remained significant in the fully adjusted model (Supplementary Table 4). In multivariable Cox regression models (*Model 2*), increased plasma levels of five proteins yielded significant associations with increased risk of mortality for study participants with AF; TIMP-4, GDF-15, GAL-3, ST-2, and MMP-3 (Table 3). None of the proteins were found to be significantly associated with re-hospitalization risk (Table 3). For participants without AF, multivariable Cox regression models (*Model 2*) showed that increased levels of TIMP-4, ST-2, MMP-2, MMP-3, and GDF-15 yielded significant associations with mortality whereas only higher levels of GDF-15 were significantly associated with re-hospitalization (Table 4).

**TABLE 3:** Cox regression analyses display associations between myocardial fibrosis biomarkers and mortality and re-hospitalization in patients with prevalent AF.

Mortality	Unadjusted			Model 1			Model 2		
	HR	95%CI	*p*	HR	95%CI	*p*	HR	95%CI	*p*
TIMP-4	1.40	1.16–1.70	4.3 × 10^–4^	1.33	1.09–1.63	0.005	1.33	1.07–1.66	**0.010**
ST2	1.44	1.20–1.74	1.3 × 10^–4^	1.47	1.21–1.79	3.9 × 10^–5^	1.48	1.18–1.85	**6.5 × 10** ^–^ **^4^**
MMP-2	1.25	1.03–1.52	0.024	–	–	–	–	–	–
MMP-9	1.07	0.89–1.29	0.471	–	–	–	–	–	–
MMP-3	1.44	1.20–1.73	8.1 × 10^–5^	1.34	1.10–1.63	0.003	1.33	1.09–1.63	**0.006**
GAL-3	1.32	1.11–1.57	0.002	1.24	1.03–1.50	0.027	1.29	1.03–1.61	**0.029**
GDF-15	1.45	1.22–1.72	2.4 × 10^–5^	1.40	1.16–1.68	4.2 × 10^–4^	1.30	1.05–1.62	**0.017**

**Re-hospitalization**	**Unadjusted**			**Model 1**			**Model 2**		
	**HR**	**95%CI**	***p***	**HR**	**95%CI**	***p***	**HR**	**95%CI**	***p***

TIMP-4	1.23	1.05–1.5	0.013	–	–	–	–	–	–
ST2	0.95	0.82–1.09	0.464	–	–	–	–	–	–
MMP-2	1	0.6–1.17	0.99	–	–	–	–	–	–
MMP-9	0.96	0.83–1.11	0.578	–	–	–	–	–	–
MMP-3	1.02	0.87–1.19	0.839	–	–	–	–	–	–
GAL-3	0.98	0.84–1.14	0.785	–	–	–	–	–	–
GDF-15	1.09	0.94–1.26	0.249	–	–	–	–	–	–

TIMP-4, Metalloproteinase inhibitor 4; ST-2, suppression of tumorigenicity 2; GAL-3, galectin-3. Matrix metalloproteinase 2, 3, and 9 (MMP-2, MMP-3, and MMP-9, respectively). Model 1: age and sex. Model 2: age, sex, body mass index, systolic blood pressure at admission, prevalence of diabetes, prior heart failure, current smoking, anticoagulation treatment, and New York heart association class (NYHA class) as independent variables. Bolded values represent significant P-values.

**TABLE 4:** Cox regression analyses displaying the associations between myocardial fibrosis biomarkers and mortality and re-hospitalization for participants without prevalent AF.

Mortality	Unadjusted			Model 1			Model 2		
	HR	95%CI	*p*	HR	95.0% CI for HR	*p*	HR	95.0% CI for HR	*p*
TIMP-4	1.39	1.11–1.74	0.004	1.33	1.02–1.74	0.035	1.32	1.01–1.74	**0.045**
ST2	1.46	1.18–1.81	4.6 × 10^–4^	1.59	1.27–1.99	6.8 × 10^–5^	1.55	1.22–1.97	**3.5 × 10** ^–^ **^4^**
MMP-2	1.46	1.14–1.85	0.002	1.52	1.16–1.99	0.002	1.45	1.10–1.91	**0.008**
MMP-9	1.19	0.92–1.54	0.179	–	–	–	–	–	–
MMP-3	1.42	1.18–1.72	2.8 × 10^–4^	1.42	1.16–1.74	7.2 × 10^–4^	1.46	1.17–1.84	**9.8 × 10** ^–^ **^4^**
GAL-3	1.36	1.09–1.70	0.008	–	–	–	–	–	–
GDF-15	1.5	1.26–1.79	5 × 10^–6^	1.63	1.30–2.04	2.5 × 10^–5^	1.56	1.22–1.99	**4.1 × 10** ^–^ **^4^**

**Re-hospitalization**	**Unadjusted**			**Model 1**			**Model 2**		
	**HR**	**95%CI**	***p***	**HR**	**95.0% CI for HR**	***p***	**HR**	**95.0% CI for HR**	***p***

TIMP-4	1.08	0.88–1.31	0.471	–	–	–	–	–	–
ST2	1.21	0.01–1.45	0.044	–	–	–	–	–	–
MMP-2	1.16	0.94–1.44	0.155	–	–	–	–	–	–
MMP-9	0.96	0.78–1.17	0.664	–	–	–	–	–	–
MMP-3	1.06	0.89–1.25	0.49	–	–	–	–	–	–
GAL-3	1.2	0.01–1.44	0.049	–	–	–	–	–	–
GDF-15	1.3	1.08–1.55	0.003	1.29	1.07–1.55	0.007	1.24	1.01–1.53	**0.039**

TIMP-4, Metalloproteinase inhibitor 4; ST-2, suppression of tumorigenicity 2; GAL-3, galectin-3. Matrix metalloproteinase 2, 3, and 9 (MMP-2, MMP-3, and MMP-9, respectively). Model 1: age and sex. Model 2: age, sex, body mass index, systolic blood pressure at admission, prevalence of diabetes, prior heart failure, current smoking, anticoagulation treatment, and New York heart association class (NYHA class) as independent variables. Bolded values represent significant *P*-values.

## Discussion

In this study, we were able to demonstrate that elevated levels of three plasma proteins previously linked to myocardial fibrosis are associated with prevalent AF in a HF population. Additionally, higher levels of five plasma proteins previously linked to myocardial fibrosis yielded an increased risk of mortality in the HF population with or without co-existing AF.

The co-existence between atrial fibrillation (AF) and heart failure has been well-established over the last decade and together they are responsible for substantial morbidity and burden on the healthcare system (1). Despite sharing common precipitating factors such as older age, hypertension, diabetes, obesity, and ischemic and non-ischemic cardiac disease, there are still limited data providing insights into the causal relationship between AF and HF. Hence, a better understanding of the pathophysiological properties of these conditions is crucial for sustainable treatment in this challenging patient population. The above-mentioned risk factors principally lead to cellular and extracellular myocardial changes such as electrophysiological and neurohormonal changes within the heart (19, 20). Furthermore, AF or HF may facilitate the progression and development of each other in several ways such as inflammatory cardiac (myocarditis and pericarditis) and non-cardiac (infections and inflammatory bowel diseases) conditions, whereas ischemic heart diseases contribute to AF/HF development due to subclinical inflammatory appearances (21–23). Evidence from large HF trials has shown that the prevalence of AF increases with the severity of HF symptoms (24) ranging from 10% in HF with NYHA classes I–II to 50% in HF with NYHA class IV. Correspondingly, studies of AF have revealed a high burden of concomitant HF with a prevalence between 21 and 68% (25). The most unfavorable hemodynamic effect linked with AF results mainly from the loss of atrial systole, ventricular chronotropic ineffectiveness, and irregular ventricular rate leading to shortened diastolic phase (26). In addition to atrial contractility loss, AF further contributes to dysfunctional ventricular filling and reduced stroke volume. The impaired atrial systolic function can precipitate HF in situations of dysfunctional ventricular filling that often accompany AF, such as mitral stenosis, ischemic heart disease, and diastolic dysfunction (27). This property together with the suboptimal electromechanical function and activation of the neurohumoral system due to reduced cardiac output creates an environment causing the deterioration of AF and HF simultaneously (28).

Increasing evidence suggests myocardial fibrosis as a crucial contributor to the cardiac remodeling seen in patients with AF (29). Myocardial fibrosis is histologically defined by a dispersed disposition of excess fibrous tissue in relation to the total mass of cardiomyocytes within the myocardial interstitium (8, 30). Replacement fibrosis is frequently initiated by cardiomyocyte death, which causes inflammatory responses and release of cytokines, chemokines, and oxidative stress, whereas in reactive fibrosis, various stimuli such as ischemia, metabolic injury, or pressure overload are responsible for the accumulation of fibrotic tissue in the absence of cell death (8, 31–33). Additionally, different cell types are involved with the fibrotic response, either directly by myofibroblast (producing fibrous tissue) or indirectly by macrophages, lymphocytes, cardiomyocytes, and mast cells (secreting profibrotic mediators). Significant accumulation of fibrotic tissue within the heart will eventually lead to left ventricular (LV) dysfunction and risk of arrhythmia (34, 35).

Although the golden standard method to assess myocardial fibrosis is through tissue biopsy, increasing evidence has shown promising links between circulating biomarkers and myocardial fibrosis. Recently Lopez et al. considered 28 plasma molecules as potential candidates for myocardial fibrosis (8). To date, only procollagen types I and III have been proven to be associated with histologically verified myocardial fibrosis (8).

### Protein biomarkers

Matrix metalloproteinases (MMPs) belong to *zinc-dependent proteolytic enzymes* and, together with tissue metalloproteinase inhibitors (TIMPs), regulate extracellular matrix (ECM) (16, 36). The balance between the synthesis and degradation process is maintained by ECM homeostasis (16). A misbalance toward MMPs results in increased fragmentation of ECM proteins, while a misbalance toward TIMP results in the protection of ECM proteins (37). High levels of both MMP-2 and TIMP-4 were demonstrated in this study, which most likely represents the counterbalance between the two proteins. Usually, profibrotic stimuli, such as proinflammatory cytokines or intensification of oxidative stress, cause a disruption in favor of the ECM protein synthesis process causing excessive fibrosis (38). Increased TIMP is suggested to be involved in ECM deposition or fibrotic processes, while reduced levels or loss of TIMP prolong the ECM degradation (39). Thus, in the normal state, TIMP can directly inhibit ECM degradation or MMP activation. Reports confirm that AF is mediated by increased activity of MMPs which has also been shown to predict HF development (40). The current study could not find any associations between AF, LAVI, and MMP-2, even though the associations were estimated as borderline significant. The lack of balance between TIMPs and MMPs could potentially be the major component of the heart remodeling process, and an area with a clear need for more evidence.

Galectin-3 is a *galactoside-binding lectin* involved in important regulatory processes such as inflammation, fibrosis, adhesion, and immunity (41). Activated macrophages secrete galectin leading to the proliferation of fibroblasts, collagen disposition following cardiac fibrosis, and myocyte disruption and potentially predispose AF (42, 43). The ARIC study (*n* = 8,436) with a median follow-up time of 16 years stated that elevated plasma galectin-3 is associated with an increased risk of incident AF (44). After adjustment for AF risk factors, participants with galectin-3 levels above the 90th percentile had a significantly higher risk of incident AF. Furthermore, studies have shown that elevated galectin-3 levels are associated with a more advanced AF, accompanied by severe comorbidities and worse outcomes (45). Even though levels of Gal-3 did not differ between participants with or without co-existent AF, higher levels of Gal-3 were associated with all-cause mortality only in participants with AF.

ST-2 belongs to the *interleukin-1 receptor family* and is released from the myocardium and vascular endothelial cells in response to pressure and hemodynamic volume overload (46). From the pathophysiological point of view, increased hemodynamic load with subsequent atrial stretch is a mechanism of AF pathophysiology and is responsible for the release of brain natriuretic peptide. Additionally, atrial stretch may lead to increased levels of ST-2. ST-2 actions within the cardiovascular system have raised questions about whether it could potentially become a novel biomarker of cardiac remodeling, myocardial infarction, HF, and AF (47). Chen et al. also pointed out that increased ST-2 levels in an AF population could be associated with an increase in the heart rate and atrial pressure compared to patients with sinus rhythm. Based on these findings, the significant association between elevated levels of ST-2 and prevalent AF demonstrated in the current study is not surprising.

Growth differentiation factor-15 (GDF-15) is a member of the transforming growth factor b cytokine superfamily and is described as a stress-responsive cytokine. Based on current evidence, it may be involved in inflammation, coagulation, oxidative stress, endothelial dysfunction, and homeostasis (48). The PARADIGM-HF trial has shown that higher levels of GDF-15 were associated with mortality and cardiovascular events in patients with HF with reduced ejection fraction (49). A two-sample Mendelian randomization study by Wang et al. was carried out using five independent large genome-wide association studies to investigate the causal association between circulating GDF-15 levels and prevalent CVD. This study provided genetic evidence, suggesting that circulating GDF-15 levels are significantly linked to the increased risk of AF, coronary artery disease, myocardial infarction, and cardioembolic stroke (50). The ARISTOTLE trail has also pinpointed that increased concentration of GDF-15 is associated with mortality, major bleeding, and stroke in a population with prevalent AF (51). Thus, GDF-15 is increased in several conditions which might explain our findings that higher GDF-15 is strongly associated with mortality also in those without prevalent AF (*p* = 4.1 × 10^–4^) and is the only protein biomarker associated with re-hospitalization.

### Strengths and limitations

As this is an observational study, no conclusions about causality can be drawn. Further, increased levels of several proteins were associated with mortality for both study sub-populations, prevalent AF vs. no AF. This might be explained by the fact that the current cohort consists of HF patients with a high burden of CV risk factors which might have affected the levels of the included proteins. Additionally, the association between higher levels of the proteins and higher mortality risk might be an adverse effect of severe HF since the vast majority belong to NYHA classes III–IV. Another limitation is the lack of complete data on echocardiographic variables, which might have affected the power needed to explore associations between proteins and left atrial volume. Blood samples were obtained after an overnight fast. It is, therefore, possible that the initial medical treatment (including furosemide) for decompensated HF might have influenced the plasma levels of biomarkers included in the CVD III panel. The collected data origin from a single regional hospital and the included participants are mainly of Caucasian descent. Hence, the applicability for other HF populations is limited.

## Conclusion

In this study, we were able to demonstrate that elevated levels of five plasma proteins previously associated with myocardial fibrosis were associated with increased mortality in a HF population with and without co-existing AF. Additionally, higher levels of four fibrosis-associated proteins were significantly associated with prevalent atrial fibrillation. Considering our findings, one may argue that we still lack an understanding of the accurate pathophysiological mechanism taking part in HF with co-existing AF. To establish this, further studies are warranted.

## Data availability statement

The raw data supporting the conclusions of this article will be made available by the authors, without undue reservation.

## Ethics statement

The studies involving human participants were reviewed and approved by the Ethical Review Board at Lund University. The patients/participants provided their written informed consent to participate in this study.

## Author contributions

ZN and HH contributed equally to writing the manuscript. AZ, AJ, AD, EB, JK, JM, MO, MM, and PP contributed equally with statistical help, data interpretation, and literature research. All authors contributed to the article and approved the submitted version.

## Footnotes

1
http://www.olink.com
