# Supplementary material for: The impact of myocardial fibrosis biomarkers in a heart failure population with atrial fibrillation—The HARVEST-Malmö study
Source: Front Cardiovasc Med. 2022 Oct 19;9:982871. doi: 10.3389/fcvm.2022.982871 (PMC9626526; doi:10.3389/fcvm.2022.982871)
Supplement: Supplementary file 1 [file Data_Sheet_1.docx]

**Supplementary material**

**Supplementary table 1.** Pearsons’s correlations analysis between the fibrosis proteins.

|  | **Pearson Correlation** | **p-value** | **95% Confidence Intervals (2-tailed)** |  |
| --- | --- | --- | --- | --- |
|  |  |  | **Lower** | **Upper** |
| **Gal-3 - GDF-15** | .344 | .000 | .242 | .437 |
| **Gal-3 - TIMP4** | .370 | .000 | .270 | .461 |
| **Gal-3 - ST2** | .277 | .000 | .172 | .376 |
| **Gal-3 - MMP-2** | .275 | .000 | .170 | .374 |
| **Gal-3 - MMP-3** | .280 | .000 | .175 | .379 |
| **Gal-3 - MMP-9** | .275 | .000 | .169 | .374 |
| **GDF-15 - TIMP4** | .411 | .000 | .315 | .499 |
| **GDF-15 - ST2** | .498 | .000 | .410 | .576 |
| **GDF-15 - MMP-2** | .576 | .000 | .497 | .645 |
| **GDF-15 - MMP-3** | .409 | .000 | .313 | .497 |
| **GDF-15 - MMP-9** | .057 | .316 | -.054 | .166 |
| **TIMP4 - ST2** | .470 | .000 | .379 | .552 |
| **TIMP4 - MMP-2** | .312 | .000 | .209 | .408 |
| **TIMP4 - MMP-3** | .316 | .000 | .213 | .412 |
| **TIMP4 - MMP-9** | .168 | .003 | .058 | .273 |
| **ST2 - MMP-2** | .353 | .000 | .252 | .446 |
| **ST2 - MMP-3** | .477 | .000 | .387 | .558 |
| **ST2 - MMP-9** | .290 | .000 | .186 | .388 |
| **MMP-2 - MMP-3** | .401 | .000 | .304 | .490 |
| **MMP-2 - MMP-9** | .011 | .846 | -.099 | .121 |
| **MMP-3 - MMP-9** | .186 | .001 | .077 | .290 |

Metalloproteinase inhibitor 4 (TIMP-4), Soluble interleukin 1 receptor-like 1 (ST-2), Matrix metalloproteinase 2,3,9 (MMP-2, MMP-3 and MMP-9 respectively), Galectin 3 (GAL-3), Growth/differentiation factor 15 (GDF-15)

**Supplementary table 2.** Logistic Regression Analysis Examining Proteins association with prevalent atrial fibrillation stratified according to gender

| **Male** | | | | | | | | | |
| --- | --- | --- | --- | --- | --- | --- | --- | --- | --- |
|  |  | | |  | | |  | | |
|  | **Unadjusted** | | | **Model 1** | | | **Model 2** | | |
| **Proteins** | **OR** | **95%CI** | **p-value** | **OR** | **95%CI** | **p-value** | **OR** | **95%CI** | **p-value** |
|  |  |  |  |  |  |  |  |  |  |
| **TIMP-4** | 2.06 | 1.47-2.91 | 3.5x10-5 | 1.76 | 1.23-2.52 | 0.002 | 1.75 | 1.14-2.67 | 0.01 |
| **ST-2** | 1.57 | 1.15-2.13 | 0.004 | 1.50 | 1.09-2.06 | 0.012 | 1.68 | 1.14-.46 | 0.009 |
| **MMP-2** | 1.42 | 1.07-1.89 | 0.017 | - | - | - | - | - | - |
| **GDF-15** | 1.51 | 1.12-2.03 | 0.006 | 1.34 | 1.0-1.8 | 0.05 | 1.41 | 0.97-2.09 | 0.083 |
| **GAL-3** | 1.27 | 0.95-1.70 | 0.102 | - | - | - | - | - | - |
| **MMP-9** | 0.80 | 0.61-1.07 | 0.132 | - | - | - | - | - | - |
| **MMP-3** | 1.29 | 0.96-1.73 | 0.92 | - | - | - | - | - | - |
| **Female** | | | | | | | | | |
|  |  | | |  | | |  | | |
|  | **Unadjusted** | | | **Model 1** | | | **Model 2** | | |
| **Proteins** | **OR** | **95%CI** | **p-value** | **OR** | **95%CI** | **p-value** | **OR** | **95%CI** | **p-value** |
|  |  |  |  |  |  |  |  |  |  |
| **TIMP-4** | 1.42 | 0.95-2.10 | 0.086 | - | - | - | - | - | - |
| **ST-2** | 1.18 | 0.82-1.72 | 0.377 | - | - | - | - | - | - |
| **MMP-2** | 1.83 | 1.17-2.89 | 0.009 | - | - | - | - | - | - |
| **GDF-15** | 1.83 | 1.12-3.01 | 0.016 | - | - | - | - | - | - |
| **GAL-3** | 0.64 | 0.41-0.99 | 0.046 | - | - | - | - | - | - |
| **MMP-9** | 0.66 | 0.44-1.01 | 0.05 | - | - | - | - | - | - |
| **MMP-3** | 1.36 | 0.88-2.09 | 0.165 | - | - | - | - | - | - |

Metalloproteinase inhibitor 4 (TIMP-4), Soluble interleukin 1 receptor-like 1 (ST-2), Matrix metalloproteinase 2,3,9 (MMP-2, MMP-3 and MMP-9 respectively), Galectin 3 (GAL-3), Growth/differentiation factor 15 (GDF-15)

Model 1: age and sex

Model 2: age, sex, body mass index, systolic blood pressure at admission, prevalence of diabetes, prior heart failure, current smoking, anticoagulation treatment and New York heart association class (NYHA-class) as independent variables

**Supplementary table 3.** Logistic Regression Analysis Examining Proteins association with left atrial volume index ≥48 ml/m^2^

| **Proteins** | **Unadjusted** | | | **Model 1** | | | **Model 2** | | |
| --- | --- | --- | --- | --- | --- | --- | --- | --- | --- |
|  | **OR** | **95%CI** | **p-value** | **OR** | **95%CI** | **p-value** | **OR** | **95%CI** | **p-value** |
| TIMP-4 | 0.91 | 0.71-1.18 | 0.489 | - | - | - | - | - | - |
| ST-2 | 0.95 | 0.73-1.24 | 0.689 | - | - | - | - | - | - |
| MMP-2 | 1.37 | 1.04-1.80 | 0.025 | - | - | - | - | - |  |
| MMP-9 | 0.81 | 0.62-1.05 | 0.109 | - | - | - | - | - | - |
| MMP-3 | 0.97 | 0.75-1.26 | 0.82 | - | - | - | - | - | - |
| GAL-3 | 0.71 | 0.54-0.93 | 0.014 | - | - | - | - | - | - |
| GDF-15 | 1.28 | 0.98-1.07 | 0.074 | - | - | - | - | - | - |

Metalloproteinase inhibitor 4 (TIMP-4), Soluble interleukin 1 receptor-like 1 (ST-2), Matrix metalloproteinase 2,3,9 (MMP-2, MMP-3 and MMP-9 respectively), Galectin 3 (GAL-3), Growth/differentiation factor 15 (GDF-15)

Model 1: age and sex

Model 2: age, sex, body mass index, systolic blood pressure at admission, prevalence of diabetes, prior heart failure, current smoking, anticoagulation treatment and New York heart association class (NYHA-class) as independent variables

**Supplementary table 4.** Cox regression analyses displaying associations between myocardial fibrosis biomarkers and mortality and re-hospitalization in the whole population (n=316)

| **Mortality** | **Unadjusted** | | | **Model 1** | | | **Model 2** | | |
| --- | --- | --- | --- | --- | --- | --- | --- | --- | --- |
|  | **HR** | **95%CI** | **p** | **HR** | **95%CI** | **p** | **HR** | **95%CI** | **p** |
| **TIMP-4** | 1.39 | 1.21-1.60 | 4x10^-6^ | 1.29 | 1.10-1.50 | 0.002 | 1.32 | 1.12-1.56 | **9.9 x 10^-4^** |
| **ST2** | 1.45 | 1.26-1.67 | 1.9x10^-7^ | 1.48 | 1.28-1.72 | 2.2x10^-7^ | 1.52 | 1.30-1.78 | **2.1 x 10^-7^** |
| **MMP-2** | 1.32 | 1.14-.53 | 1.9x10^-4^ | 1.26 | 1.08-1.45 | 0.003 | 1.25 | 1.07-1.47 | **0.006** |
| **MMP-3** | 1.43 | 1.25-1.63 | 8.5x10^-8^ | 1.36 | 1.18-1.57 | 2.1x10^-5^ | 1.38 | 1.19-1.59 | **1.5x10^-5^** |
| **GAL-3** | 1.34 | 1.17-1.54 | 4.5x10^-5^ | 1.29 | 1.12-1.50 | 5.9 x10^-4^ | 1.27 | 1.08-1.50 | **0.003** |
| **MMP-9** | 1.09 | 0.95-1.28 | 0.218 | - | - | - | - | - | **-** |
| **GDF-15** | 1.46 | 1.29-1.64 | 1.2 x10^-9^ | 1.41 | 1.23-1.62 | 1 x10^-6^ | 1.39 | 1.19-1.63 | **7.4 x10^-4^** |
| **Re-hospitalization** | **Unadjusted** | | | **Model 1** | | | **Model 2** | | |
|  | **HR** | **95%CI** | **p** | **HR** | **95%CI** | **p** | **HR** | **95%CI** | **p** |
| **TIMP-4** | 1.20 | 1.06-1.35 | 0.004 | 1.20 | 1.05-1.38 | 0.009 | 1.16 | 1.01-1.33 | **0.046** |
| **ST2** | 1.07 | 0.96-1.20 | 0.205 | - | - | - | - | - | - |
| **MMP-2** | 1.09 | 0.97-1.23 | 1.149 | - | - | - | - | - | - |
| **MMP-9** | 0.94 | 0.84-1.06 | 0.335 | - | - | - | - | - | - |
| **MMP-3** | 1.06 | 0.95-1.18 | 0.320 | - | - | - | - | - | - |
| **GAL-3** | 1.08 | 0.96-1.20 | 0.215 | - | - | - | - | - | - |
| **GDF-15** | 1.20 | 1.07-1.33 | 0.001 | 1.18 | 1.05-1.33 | 0.005 | 1.09 | 0.96-1.23 | 0.193 |

Metalloproteinase inhibitor 4 (TIMP-4), Soluble interleukin 1 receptor-like 1 (ST-2), Galectin 3 (GAL-3) and Matrix metalloproteinase 2, 3 and 9 (MMP-2, MMP-3 and MMP-9 respectively)

Model 1: age and sex

Model 2: age, sex, body mass index, systolic blood pressure at admission, prevalence of diabetes, prior heart failure, current smoking, anticoagulation treatment and New York heart association class (NYHA-class) as independent variables
